# Cytokeratin-18 and uric acid predicts disease severity in Taiwanese nonalcoholic steatohepatitis patients

**DOI:** 10.1371/journal.pone.0174394

**Published:** 2017-05-04

**Authors:** Jee-Fu Huang, Ming-Lun Yeh, Chung-Feng Huang, Ching-I Huang, Pei-Chien Tsai, Chi-Ming Tai, Hua-Ling Yang, Chia-Yen Dai, Meng-Hsuan Hsieh, Shinn-Chern Chen, Ming-Lung Yu, Wan-Long Chuang

**Affiliations:** 1 Hepatobiliary Division, Department of Internal Medicine, Kaohsiung Medical University Hospital, Kaohsiung, Taiwan; 2 Graduate Institute of Clinical Medicine, Kaohsiung Medical University, Kaohsiung, Taiwan; 3 Faculty of Internal Medicine, College of Medicine, Kaohsiung Medical University, Kaohsiung, Taiwan; 4 Department of Internal Medicine, E-Da Hospital, I-Shou University, Kaohsiung, Taiwan; Chang Gung Memorial Hospital Kaohsiung Branch, TAIWAN

## Abstract

**Background & aims:**

Identification of disease severity remains a challenge in the management of non-alcoholic steatohepatitis (NASH). Cytokeratin-18 (CK18), is a recently developed non-invasive biomarker for NASH. We aimed to assess the performance of CK18 in disease severity prediction among Taiwanese NASH patients.

**Methods:**

A total of 76 biopsy-proven NASH patients (54 males, age = 41.0 ± 13.5 years) were consecutively recruited. The optimal cutoff values of CK18 for each stage of fibrosis were correlated with their histopathological manifestations.

**Results:**

There were 23 (30.3%) patients of Metavir fibrosis stage 0 (F0), 32 (42.1%) patients of F1, 14 (18.4%) patients of F2, and 7 (9.2%) patients of F3-4, respectively. The CK18 levels among those patients of F0, F1, F2, F3-4 were 86.7 ± 75.6 U/L, 122.4 ± 123.8 U/L, 160.7 ± 120.4 U/L, and 507.3 ± 343 U/L, respectively (trend for P<0.001). The adjusted optimal cutoff value for F2 prediction was 312.5 U/L, yielding the sensitivity, specificity, positive predictive value (PPV), negative predictive value (NPV), and the accuracy of 96.4%, 28.6%, 77.9%, 75%, and 77.6%, respectively (P = 0.009). For the prediction of advanced fibrosis (F3-4), the adjusted optimal cutoff value was 374.5 U/L, yielding the sensitivity, specificity, PPV, NPV, and the accuracy of 97.1%, 54.1%, 95.7%, 66.7%, and 77.6%, respectively (P = 0.003). Among those patients without hyperuricemia, the PPV, NPV, and accuracy of CK18 reached 100%, 95.8%, and 96%, respectively (P<0.001).

**Conclusions:**

CK18 combined with uric acid measurement is a promising non-invasive biomarker for prediction of disease severity in NASH patients.

**Trial registration:**

ClinicalTrials.gov NCT01068444.

## Introduction

In addition to viral hepatitis infection, the importance of non-alcoholic fatty liver disease (NAFLD) has progressively been emphasized in recent decades globally. Non-alcoholic steatohepatitis (NASH), as an extreme form of NAFLD, is characterized by the presence of significant necroinflammation and/or fibrosis development histopathologically. It is a progressive liver disease in nature and can lead to cirrhosis and hepatocellular carcinoma (HCC), especially in patients with older age, obesity and diabetes mellitus (DM) [1–5]. NASH is basically a hepatic manifestation of metabolic syndrome (MetS) and has a close link with other metabolic disorders, such as obesity, dyslipidemia, hypertension and DM [6]. The scenario of a higher overall mortality due to cardiovascular events as compared with controls has made it a critical global issue. In addition, Asians are more susceptible to NASH as well as metabolic disorders than other ethnicities [7,8]. Therefore, the features and characteristics of NASH deserve to be further elucidated in Asia [9].

Early identification of disease progression before the development of fibrosis is quite challenging in the management of NASH. Although liver biopsy is the gold standard of diagnosis, the invasiveness and potential complications much limits the clinical application. Therefore, the development of non-invasive methods aiming to assess the disease severity has been vigorously investigated in the past decade.

Cytokeratin-18 (CK18), which is generated during cell death or apoptosis, is a recently developed serum biomarker for NASH diagnosis [10]. Following the apoptotic cell death of injured hepatocytes due to obesity-related damage, caspase-cleaved CK18 fragments enter the bloodstream. The presence of CK18 fragments in the blood differentiates NASH from simple steatosis since such apoptotic activity does not occur in the condition of simple steatosis. Previous study in adults indicated that every 50 U/L increase in the plasma level of CK18 increases the likelihood of NASH by 30% [11]. The observation was further extended to the children, demonstrating that for every 10 U/L increase in CK18 levels, the likelihood of having NASH increased by 70% after adjusting for multiple confounders [12]. CK18 has also been demonstrated to be the most accurate biomarker for NASH/NAFLD diagnosis [13].

Consequently, we conducted the current study from a prospective NASH cohort in Taiwan. We aimed to explore the performance of CK18 in the disease prediction among Taiwanese NASH patients. We also aimed to elucidate the extent of interaction between CK18 and other metabolic abnormalities.

## Methods

### Study design

This prospective, multi-centre study was conducted in one medical centre and 2 regional core hospitals in Taiwan from January 2007 to July 2011. The ethical committee of the Kaohsiung Medical University Hospital approved the study and the study has been conducted according to the Declaration of Helsinki. Written informed consent for interview, anthropomorphic measurements, blood sampling, and medical record review were obtained from patients prior to enrollment. All subjects underwent a 12-h overnight fast before blood tests, which included CK18, fasting plasma glucose (FPG), insulin, total cholesterol (TC), high-density lipoprotein cholesterol (HDL-C), low-density lipoprotein cholesterol (LDL-C), triglycerides (TG), uric acid (UA), aspartate aminotransferase (AST) and alanine aminotransferase (ALT) levels. In addition, anthropometric data, which included blood pressure, waist circumference, body weight and height, were measured using standardized techniques. For those without known DM in their past history, they first received a 75-g oral glucose tolerance test (OGTT) and then 2-hour post load plasma glucose level was measured.

### Patient selection

#### Inclusion criteria

Eligible patients were treatment-naive Taiwanese patients, aged 18–65 years, who satisfied all of the following inclusion criteria were eligible to participate: (1) had undergone a liver biopsy within 6 months before entry, the results of which were consistent with NASH, i.e. a combination of steatosis (>5% steatosis), hepatocellular injury and inflammation; (2) displayed an increased serum ALT level, defined as >1.5 times the upper limit of the normal range for at least two measurements within 6 months preceding the study entry; and (3) ethanol consumption of < 20 g/day.

#### Exclusion criteria

Patients were excluded from the study if any of the following criteria existed: (1) laboratory or histologic findings highly suggestive of liver disease of other etiologies, such as autoimmune hepatitis, primary biliary cirrhosis, biliary obstruction, hemochromatosis, alpha-1-antitrypsin deficiency or Wilson’s disease.; (2) ALT or AST levels greater than 10 times the normal; (3) abnormal total bilirubin or albumin level, prolonged prothrombin time, or platelet count below the lower limit of normal; (4) decompensated cirrhosis (Child–Pugh class B or C) or overt hepatic failure; (5) Treatment with any drugs known to cause hepatic steatosis (i.e., corticosteroids, high-dose estrogens, methotrexate, amiodarone, calcium channel blockers, spironolactone, sulfasalazine, naproxen, or oxacillin) within 6 months prior to the study; (6) psychiatric condition, previous liver transplantation, or evidence of HCC.

### Laboratory analyses

FPG, TC, HDL-C, LDL-C, TG, UA, AST and ALT levels were measured on a multichannel autoanalyzer (Hitachi Inc, Tokyo, Japan). Serum was analysed for the M30 fragment of CK18 by ELISA (Peviva AB, Bromma, Sweden) according to the manufacturer’s recommendations. All assays were performed in duplicates. Fasting serum insulin levels were measured by radioimmunoassay (Diagnostic Products Co., Los Angeles, CA).

MetS was defined based on the updated National Cholesterol Education Program Adult Treatment Panel III criteria for Asian-Americans, modified by the criteria of obesity proposed for Asians by the Steering Committee of the Regional Office for the Western Pacific Region of WHO as presenting at least three of the following components: 1) waist circumferences >90 cm in men or >80 cm in women; 2) TG >150 mg/dL; 3) HDL-C < 40 mg/dL in men or < 50 mg/dL in women; 4) blood pressure >130/85 mmHg or current use of antihypertensive medications; or 5) FPG>100 mg/dL or on oral anti-diabetic agents or insulin.

IR was calculated on the basis of FPG and insulin levels, according to the homeostasis model assessment (HOMA) method [14]. The formulas for the HOMA-IR = FPG (mg/dL) × fasting insulin level (*μ*U/mL)/405.

### Histological analyses

For each patient, a liver biopsy specimen of at least 2 cm in length was taken and fixed in 10% formalin buffer. Biopsy samples were stained with hematoxylin-eosin and the results were then reported by a dedicated liver pathologist blinded to each patient and who is experienced with the NASH.

*Steatosis*. The extent of hepatic steatosis was graded according to the area occupied by that fatty hepatocytes on light microscopy; none (0–5%), mild (5–33%), moderate (33–66%) and severe (>66%) [15]

#### Histological grading and staging

Histological grading of NASH was made based on histological activity index (HAI) by Knodell et al [16]. The NAFLD Activity Score (NAS) was also assessed on the individual scores for steatosis (0–3), inflammation (0–3), and ballooning (0–2) by the scoring system[15]. Fibrosis score for steatohepatitis is determined with the staging from F0 to F4 according to Metavir classification [17].

### Statistical analyses

Frequency was compared between groups using the χ^2^ test, with the Yates correction, or Fisher’s exact test. Results are expressed as mean values ± standard deviation (SD) and were compared between groups using analysis of variance and the Student’s t test, or nonparametric Mann–Whitney U test when appropriate. Attempts were made to derive the optimal cutoff values of CK18 level that would best predict different stages of fibrosis by choosing the point on the receiver operating characteristic curve appropriately. All statistical analyses were based on two-sided hypothesis tests with a significance level of p<0.05. Quality control procedures, database processing, and analyses were performed using the SPSS 12.0 statistical package (SPSS Inc., Chicago, IL, USA).

## Results

A total of 76 biopsy-proven NASH patients (54 males, age = 41.0 ± 13.5 years) were consecutively recruited. Their demographic characteristics were shown in Table 1. Forty-one (53.9%) patients had DM and 49 (64.5%) had hypertension. There were 16 (21.1%) non-obese patients with their BMI less than 25 kg/m^2^. Among 60 obese patients, 46 (76.7%) were males, which was substantially higher than their non-obese counterparts (50%) (P = 0.06).The obese patients had a higher BMI, a higher HOMA-IR, a higher UA level, a higher steatosis and ballooning grades, and a higher NAS compared with their non-obese counterparts. There was no significant difference of CK18 level between obese and non-obese groups.

**Table 1:** Characteristics of the NASH patients.

Characteristic	Total	Obese	Non-obese	
	N = 76	N = 60	N = 16	P
Age (years)	41 ± 13.5	40 ± 13.5	44.9 ± 13.2	0.19
Male, n (%)	54 (71.1)	46 (76.7)	8 (50)	0.06
BMI (kg/m^2^)	28.7 ± 4.4	30.1 ± 3.8	23.4 ± 1.5	<0.001
ALT (IU/L)	117 ± 87.9	121.4 ± 96.6	100.3 ± 40	0.4
AST (IU/L)	63.1 ± 33.3	63.7 ± 34.3	61.1 ± 30.2	0.78
A1c (%)	6.3 ± 1.0	6.3 ± 1.0	6.3 ± 1.0	0.99
Fasting glucose level (mg/dL)	106.9 ± 27.0	107.6 ± 26.5	104 ± 29.4	0.64
Fasting insulin level (*μ*U/mL)	12.8 ± 9.1	13.8 ± 9	9.1 ± 8.5	0.07
HOMA-IR	3.4 ± 2.8	3.7 ± 2.9	2.1 ± 1.5	<0.001
Total Cholesterol (mg/dL)	205.3 ± 37.4	206.3 ± 36.1	201.6 ± 42.8	0.66
HDL-C (mg/dL)	45.9 ± 24.2	46.3 ± 26.4	44.3 ± 13.8	0.77
TG (mg/dL)	164.9 ± 71.7	161.6 ± 67	177.3 ± 88.6	0.44
Diabetes, n (%)	41 (53.9)	31 (51.7)	10 (62.5)	0.58
Hypertension, n (%)	49 (64.5)	40 (66.7)	9 (56.3)	0.56
Uric acid (mg/dL)	6.6 ± 1.7	6.8 ± 1.7	5.8 ± 1.3	0.03
Albumin (g/dL)	4.4 ± 0.3	4.4 ± 0.3	4.4 ± 0.4	0.69
Platelet count (mm^3^)	235.9 ± 57	239.7 ± 59.7	221.4 ± 44	0.26
rGT (U/L)	64.1 ± 56	60.3 ± 37.3	77.8 ± 99.3	0.27
Ferritin (ng/mL)	328.1 ± 232.6	338.5 ± 234.7	289.3 ± 227.8	0.46
Apelin	2.7 ± 1.5	2.6 ± 1.3	2.8 ± 2.2	0.7
CK-18 Log_10_	2.3 ± 0.7	2.3 ± 0.7	2.2 ± 0.7	0.59
HAI	5.9 ± 2.5	5.9 ± 2.6	6.0 ± 2.2	0.93
Steatosis score	2.5 ± 0.7	2.6 ± 0.6	2.1 ± 0.7	0.002
Ballooning score	1.8 ± 0.7	1.9 ± 0.8	1.4 ± 0.5	0.01
Fibrosis, *n* (%)				0.49
F0-1	55 (72.4)	42 (70)	11 (68.8)	
F2	14 (18.4)	13 (21.7)	2 (12.5)	
F3–4	7 (9.2)	5 (8.4)	3 (18.8)	
NAS	5.3 ± 1.7	5.6 ± 1.7	4.3 ± 1.3	0.006

Values expressed as mean ± standard deviation. Parenthesis indicates percentage. NASH, non-alcoholic steatohepatitis; BMI, body mass index; ALT, alanine aminotransferase; AST, aspartate aminotransferase; HOMA-IR, homeostasis model assessment of insulin resistance; HDL-C, high-density lipoprotein cholesterol; TG, triglycerides; γGT, γ-glutamyl transferase; HAI, histological activity index; NAS, non-alcoholic steatohepatitis activity score.

The HAI and the NAS among the patients were 5.9 ± 2.5, and 5.3 ± 1.7, respectively. There were 23 (30.3%) patients of F0, 32 (42.1%) patients of F1, 14 (18.4%) patients of F2, and 7 (9.2%) patients of F3-4, respectively. There was no significant difference of fibrosis stage distribution between obese and non-obese patients. The CK18 levels among those patients of F0, F1, F2, F3-4 were 86.7 ± 75.6 U/L, 122.4 ± 123.8 U/L, 160.7 ± 120.4 U/L, and 507.3 ± 343 U/L, respectively (trend for P<0.001). There was no significant difference of CK18 levels in different extents of steatosis, ballooning and lobular inflammation. No significant correlation between CK18 and NAS was observed.

The optimal cutoff values of CK18 were calculated among 76 NASH patients to maximize sensitivity and specificity for the prediction of the presence of early fibrosis development (F1), significant fibrosis (F2), and advanced fibrosis (F3-4), respectively (Table 2). By adjusting age, sex, BMI, TC, TG and IR, the optimal cutoff value for prediction of F1 was 62.5 U/L. The sensitivity, specificity, positive predictive value, negative predictive value, and the accuracy were 60.9%, 64.2%, 42.4%, 79.1%, and 63.2%, respectively (P = 0.048). The optimal cutoff value for F2 prediction was 312.5 U/L. The sensitivity, specificity, positive predictive value, negative predictive value, and the accuracy were 96.4%, 28.6%, 77.9%, 75%, and 77.6%, respectively (P = 0.009). For the prediction of advanced fibrosis (F3-4), the optimal cutoff value was 374.5 U/L, yielding the sensitivity, specificity, positive predictive value, negative predictive value, and the accuracy were 97.1%, 54.1%, 95.7%, 66.7%, and 77.6%, respectively (P = 0.003). Among those patients without hyperuricemia (defined as <7 mg/dL for men and <6.0mg/dL for women), the PPV, NPV, and accuracy of CK18 could reach 100%, 95.8%, and 96%, respectively (P< 0.001) (Table 3).

**Table 2:** The optimal cutoff values and performance characteristics of CK18 for fibrosis stages.

	Optimal cutoff value* (U/L)	Sensitivity (%)	Specificity (%)	PPV (%)	NPV (%)	Accuracy (%)	Adjusted p value
F1	62.5	60.9	64.2	42.4	79.1	63.2	0.048
F2	312.5	96.4	28.6	77.9	75	77.6	0.009
F3-4	374.5	97.1	54.1	95.7	66.7	93.4	0.003

*Adjusted for age, sex, body mass index, total cholesterol, triglycerides, insulin resistance.

PPV, positive predictive value; NPV, negative predictive value.

**Table 3:** The performance characteristics of combined CK18 level and the absence of hyperuricemia for fibrosis stages.

	Optimal cutoff value* (U/L)	Sensitivity (%)	Specificity (%)	PPV (%)	NPV (%)	Accuracy (%)	Adjusted p value
F1	<62.5/UA-	32.7	95.2	94.7	35.1	50.0	0.016
F2	<312.5/UA-	47.8	100	100	16.3	52.6	0.017
F3-4	>374.5/UA-	57.1	100	100	95.8	96.0	<0.001

*Adjusted for age, sex, body mass index, total cholesterol, triglycerides, insulin resistance. UA-: absence of hyperuricemia.

## Discussion

NASH is a growing liver disease worldwide with the majority of the patients suffered from associated metabolic disorders such as obesity, DM, dyslipidemia, and hypertension. With the introduction of westernized lifestyle and the increasing frequency of obesity in the Asia-Pacific region, the prevalence of NAFLD/NASH has rapidly increased over the past decades [18]. Therefore, to identify those victims of fibrosis development for the implementation of stringent interventional strategies and surveillance of liver disease complications remains to be a challenging task. Our study demonstrated that CK18, as a non-invasive biomarker, had a significant expression correlated with fibrosis stages. Meanwhile, our results showed that the performance of CK18 was excellent in predicting advanced fibrosis with accuracy of more than 90%, particularly in those without hyperuricemia. It therefore provided the reference evidence showing CK18 is a promising non-invasive tool for disease severity assessment in NASH patients.

The inherent disadvantages of liver biopsy, including invasiveness, risk of life-threatening complications, intra- and inter-observer variability, and sampling error, have much limited its clinical application. In addition to sonographic-based methods, serum biomarkers have been explored in the past decade. From the view of pathogenic mechanisms, Mallory bodies and hepatocyte ballooning are two important hallmarks of NASH. The features are closely associated with disease severity and progression. When hepatocytes are chronically exposed to oxidative stress subsequent to NASH, the features of ballooning, fat accumulation, and a disruption in the keratin develop. CK18 is a keratin-containing protein involved in cytoskeleton cell formation. During hepatocyte apoptosis, it is cleaved by caspases and released in circulation as fragments, which are easily detectable and used as markers of apoptosis to identify patients with NASH [19].

Previous studies have indicated that CK18 was able to distinguish steatohepatitis from hepatic steatosis in a clinical setting [20,21]. The concordant results were also proved in patients of morbid obesity, pediatric patients and even chronic hepatitis C patients [12,22,23]. Liver fibrosis is the final result of a wide variety of types of liver injury. The current study extended the observation showing the competent performance of CK18 in the prediction of fibrosis. The results were echoed previous studies from Asia showing the good performance of CK18 in the diagnosis of NASH [13,24,25]. However, the somewhat discordant cutoff values between studies await elusive and collaborative study across races and regions. In addition, the discrimination performance of CK18 in the early stages of fibrosis development, namely F1, did not meet the level of excellence. It may be the subtle and minute changes of keratin disruption during the early stages of fibrosis formation and/or inflammation. Further incorporation with other biomarkers or non-invasive tools deserves to be explored. The other explanation may be that the fibrosis staging of NASH in Asians has been reported to be relatively milder than that of Westerns [3,26,27]. Meanwhile, the presence of early fibrosis was not significantly different between obese and non-obese patients [8]. Whether genetic background plays a role in it also needs elucidation.

Our previous study has demonstrated that there was a significant inverse correlation between UA level and fibrosis stages. Therefore, the absence of hyperuricemia was predictive of significant fibrosis at least in Asians. The current study extended the observation that advanced fibrosis could be accurately diagnosed by CK18 level in the absence of hyperuricemia. Whether UA is a protective or a risk factor in this disorder of underlying metabolic abnormality awaits to be investigated. UA carries an antioxidant protective response against oxidative stress and UA contributes to > 50% of the antioxidant capacity of the blood [28]. The exact role and whether UA is a protective factor in the disease progression of NASH remain to be elusive. The issue needs to be determined across different ethnicities, nutritional status and associated metabolic disorders in the future collaborative study. Another limitation of the study was that the mutual interaction between CK18 and UA remains to be further investigated. The recruitment of more patients without hyperuricemia for further analysis could provide informative evidence in this field.

In conclusion, the current study, on a biopsy-proven cohort, demonstrated that CK18 is a promising non-invasive biomarker for prediction of disease severity in Taiwanese NASH patients. Further improvement of the performance by incorporated with other non-invasive methods deserves to be investigated. With the blooming of various therapeutic interventions for the management of NASH recently, a longitudinal follow-up observation is needed for validation of CK18 in this aspect.
